# Risk prediction model of impacted supernumerary tooth-associated root resorption in children based on cone-beam computed tomography analysis: a case control study

**DOI:** 10.1186/s12903-024-04493-2

**Published:** 2024-08-09

**Authors:** Yakang Li, Yuanmin Zhang, Linpei Gao, Menghao Lyu, Baozhu Zheng, Fengqiu Zhang, Junji Xu

**Affiliations:** 1 Department of Periodontics, School of Stomatology, Capital Medical University, Tian Tan Xi Li No.4, Beijing, 100050 China; 2 Department of Pediatric Dentistry, School of Stomatology, Capital Medical University, Beijing, China; 3 Beijing Key Laboratory of Tooth Regeneration and Function Reconstruction, School of Stomatology, Capital Medical University, Beijing, China; 4 Beijing Laboratory of Oral Health, Capital Medical University, Beijing, China; 5 College & Hospital of Stomatology, Key Lab. of Oral Diseases Research of Anhui Province, Anhui Medical University, Hefei, Anhui China

**Keywords:** Cone-Beam Computed Tomography, Root resorption, Supernumerary tooth

## Abstract

**Background:**

External surface resorption is pressure-induced resorption and occurs on the external surface of the root, pressure exerted by impacted teeth, is common causes of external surface resorption. Predictive risk factors of impacted supernumerary tooth-associated root resorption (ISTARR) mentioned in this article include supernumerary teeth and patient factors. To investigate the risk factors of impacted supernumerary tooth-associated root resorption and predict the incidence of root resorption.

**Methods:**

This restrospective study enrolled 324 patients with impacted supernumerary tooth. All Cone-Beam Computed Tomography (CBCT) data and patient information were divided into two groups (without tooth root resorption and with root resorption). CBCT images and patient information (age and gender) of 133 patients had adjacent tooth root resorption and 191 did not. seven variables were analysed using binary logistic regression.

**Results:**

Individual analysis of potential risk factors showed that age, crown mesiodistal direction, root formation, and odontotheca of the impacted supernumerary tooth were associated significantly with ISTARR. Binary logistic regression showed that impacted supernumerary tooth with odontotheca (Odd Ratio = 2.926), the crown is in the middle (Odd Ratio = 1.446), located at the middle third of the adjacent tooth root (Odd Ratio = 1.614), complete root development (Odd Ratio = 1.334), and patient’s age (Odd Ratio = 1.261) were significantly associated with ISTARR risk.

**Conclusions:**

The risk factors of root resorption can be detected and predicted early according to the features of supernumerary tooth and patient’s age. Still, more prospective studies with larger sample size are needed to validate the result.

**Supplementary Information:**

The online version contains supplementary material available at 10.1186/s12903-024-04493-2.

## Background

Supernumerary tooth is a developmental abnormality of tooth number with an incidence of 0.3–3.5% [1]. McBeain et al. [2] reported most cases occur in the anterior maxillary region (51.2%). Most superabundant teeth fail to erupt or even are completely ambushed. Impacted supernumerary teeth may cause dentition disorder, and increase the chance of follicular cyst formation and recurrent infections. Moreover, impacted supernumerary teeth vary greatly in inclination and location and can lead to resorption of neighbouring incisors, irreversible damage that can potentially end in teeth loss [3, 4].

Impacted supernumerary tooth-associated root resorption (ISTARR) tends to be diagnosed late because of a general lack of symptoms and clinician reliance on two-dimensional radiographic imaging for diagnosis. Image enlargement, distortion, and structure overlap are common limitations of two-dimensional radiographic techniques that reduce image quality and diagnostic accuracy [2, 5–7]. Advances in cone-beam (CB) computed tomography (CT) techniques have significantly improved the sensitivity and accuracy of the diagnosis of root resorption. Based on the CT technique, root resorption can be found in about 25.37% ~ 40% of the incisors adjacent to impacted tooth [8, 9]. 

Every patient has its own supernumerary tooth location and potential risk factors for ISTARR, studies evaluating potential risk factors for ISATRR in - tooth impacted patients have identified various features related to root resorption. For example, patient gender (female) has been reported as a risk factor [10, 11]. Heboyan et al. [12] reported that stimulation of the osteoclast formation (resorption cells) may be genetically linked. However, other studies reported there is no significant relationship between the occurrence of resorption and gender [13, 14]. Most other studies based on two-dimensional panoramic imaging have pointed to local features, for example, the location, direction, and height of supernumerary tooth, the development of tooth roots, and condition of dental cysts, especially those directly associated with the impacted supernumerary tooth [5]. Lai et al. [15] reported specific impacted tooth location has been suggested to increase root resorption by 50%. However, several recent studies based on three-dimensional CT techniques have emphasized the role of physical pressure or contact from the impacted tooth [16]. Moreover, CBCT evaluation, which may be influenced by training and practicing experience, is subjective and may have minor variation during orthodontic practice. As a result, predictors for clinicians evaluating the risk of adjacent tooth root resorption are needed.

In this retrospective study, we use three-dimensional CBCT images to analyse the potential risk factors for impacted supernumerary tooth-associated root resorption (ISTARR). Age and gender, together with several local factors involving the location, direction, depth, developmental stage, and follicle size are assessed as potential risk factors and significant predictors for ISTARR were identified.

## Methods

### Study design and patient selection

This single -centred case-control study follows the STROCSS 2021 guidelines. 324 (4-13years old) cases with CBCT images and general patient information were from Department of Children’s Stomatology, Beijing Stomatological Hospital, Capital Medical University from June 2021 to December 2022 for various reasons, such as impacted teeth, retention of deciduous and abnormal number of teeth. Sample inclusion criteria are (1) age 4 to 13 years at the time of the CT scan; (2) presence of supernumerary tooth impaction; (3) no previous orthodontic treatment; (4) no history of dental trauma; (5) all cases are non-syndromic cases and without systemic diseases, discovered as incidental findings during routine radiological examinations; (6) no history of anterior maxillary dental surgery. All study procedures were reviewed and approved by the ethics committee before the study was conducted, all patients’ parents agree for CBCT images using authority.

### CBCT image analysis

In CBCT examination, conical beam CT Kodak 9000 3D CBCT (Dental Systems, Carestream Health, Rochester, USA) was used for scanning. The patient sat upright in the projection chair, and the chin was placed on the jaw support, so that the bisector line of the Angle between the auditory canthus and the auditory body line was parallel to the ground. The median sagittal plane of the patient was overlapped with the sagittal laser positioning line of the machine. Ask the patient to hold the handle and keep the upper and lower dentition in the median occlusal position. Scanning condition: 70 kV; 10 mA; 10.8s; Voxel 76 μm*76µm *76µm, using 360degree rotation projection.

The CBCT images were read by three experienced radiologists with at least 3 years of experience with CBCT in the same slice. The unified standard for root resorption is the presence of root surface defects with a depth greater than 1 mm in the horizontal and sagittal positions of CBCT images. Before evaluation, the examiner participated in calibration training. 20% of the sample was selected randomly and evaluated by the examiner and an endodontist (with more than 5 years of endodontic experience). The inter-observer agreement was then determined by calculating the kappa value which was 79.6%. Cases of disagreement were evaluated and discussed by the observers concurrently until a final agreement was reached. Two weeks after the evaluation, a second analysis was performed blindly by the same examiner using approximately 20% of the sample to assess the intra-observer reliability. The difference between the first and the second observations was statistically non-significant. All analysis performed on the same monitor with the 3D Imaging software.

Detailed characterizations of supernumerary tooth in 3-dimentional CBCT radiography.

1. Location of crown (buccal-lingual position in the sagittal plane): labial/buccal, both/within arch, and palatal/lingual.
2. Direction of supernumerary tooth (direction of the crown on the coronal plane): mesial, middle, distal, horizontal and inverted.
3. Height of supernumerary tooth: high (located in the neck of adjacent tooth), median (located in the middle of the adjacent tooth’s root) and low (located near the root apex of the adjacent tooth).
4. Odontotheca (hypodense shadow around the tooth): Yes or No.
5. Complete root formation (image of root development and apical foramen closure): Yes or No.

### Statistical analysis

The R language GGally package was used to conduct matrix description of the correlation of data parameters, and SPSS 27.0 statistical software was used to conduct non-parametric statistics for clinical indicators (Wilcox rank sum test for age, chi-square test for gender, location, crown direction, root formation and odontotheca) [17]. The sample size was calculated using G*Power software, with a minimum sample size of 220. The sample size of this case is 324, so it meets the requirements. R language car and rms packages were used for binary Logistic regression analysis of all data, and Nomogram graph was made to show the prediction model. *P* < 0.05 was considered statistically significant.

## Results

**Fig. 1:**
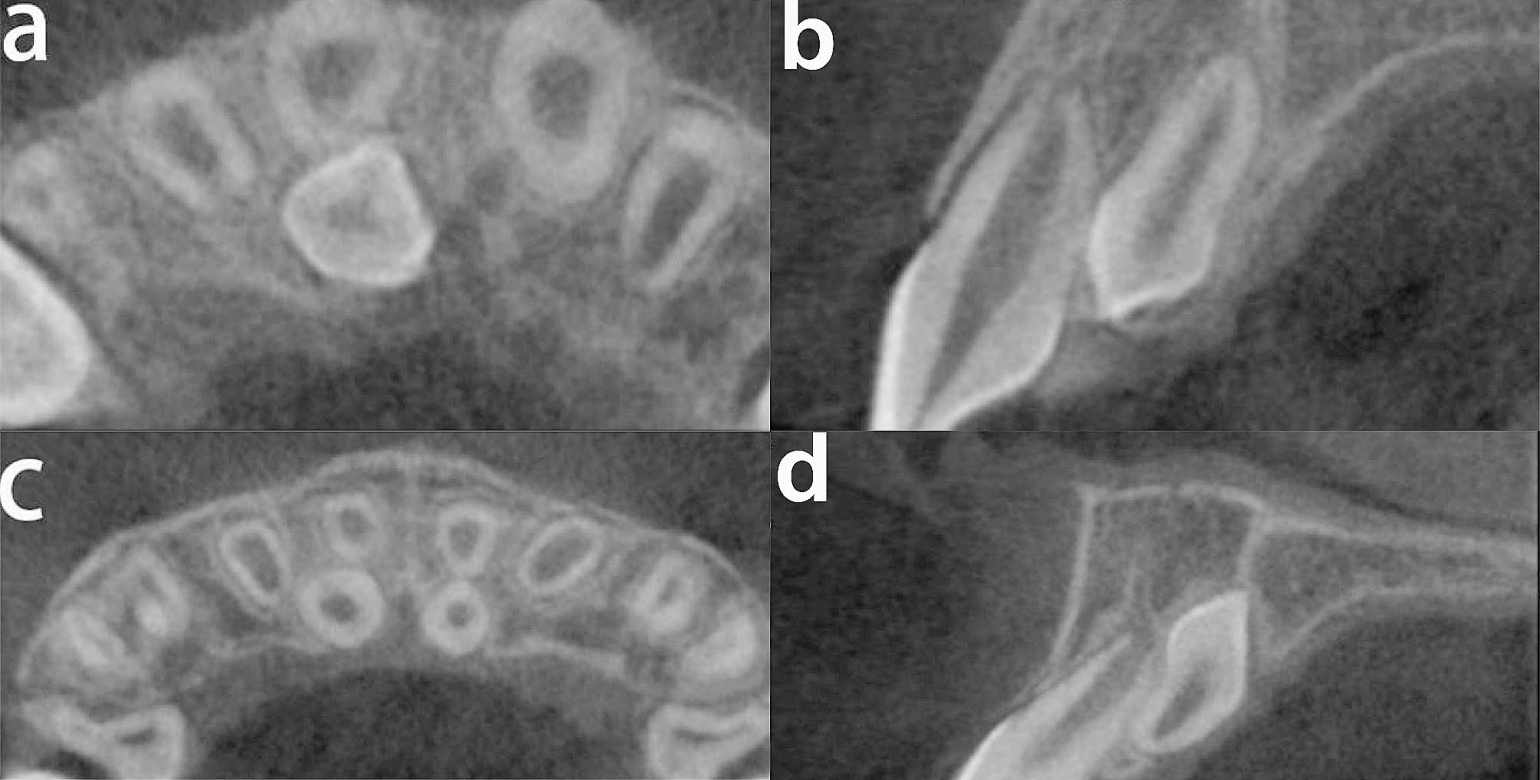
Cross and sagittal-sectional CBCT images illustrating presence of adjacent tooth roots resorption caused by supernumerary teeth. CBCT reveals the supernumerary teeth impacted on the 11 resulting in disappearance of the periodontal ligament in this region and root surface defects with a depth greater than 1 mm in the horizontal and sagittal positions (**a**, **b**). the supernumerary teeth impacted on the 11and 21 without resulting in root resorption, and appearance of the periodontal ligament in this region (**c**, **d**)

A total of 133 adjacent tooth (104males, 29 females) among the 324 cases (139males, 85females) of supernumerary tooth impaction were resorbed, resulting in a resorption rate of 41%. Root absorption of adjacent tooth caused by the patient’s impacted supernumerary tooth was shown by CBCT (Fig. 1). Individual results for each variable are shown in(Tables 1 and 2).

**Table 1:** Descriptive and inferential statistics of relationship between variables such as gender, position, direction, etc. and root resorption

		Without root resorption	Root resorption	χ^2^	*p*-value*
Gender	Female	56	29		
	Male	135	104	2.288	0.158
Location	Labial	10	2		
	Palatal	132	100		
	Both	49	31	3.527	0.172
Direction	Mesial	10	16		
	Middle	30	8		
	Distal	15	23		
	Horizontal	17	7		
	Inverted	119	79	18.255	0.001
Height	Low	24	20		
	Median	35	50		
	High	132	63	17.608	0.001
Root formation	No	125	63		
	Yes	66	70	10.519	0.001
Odontotheca	No	63	19		
	Yes	128	114	14.502	0.001

* Pearson chi-square test P-value (significance at *p* < 0.05)

**Fig. 2:**
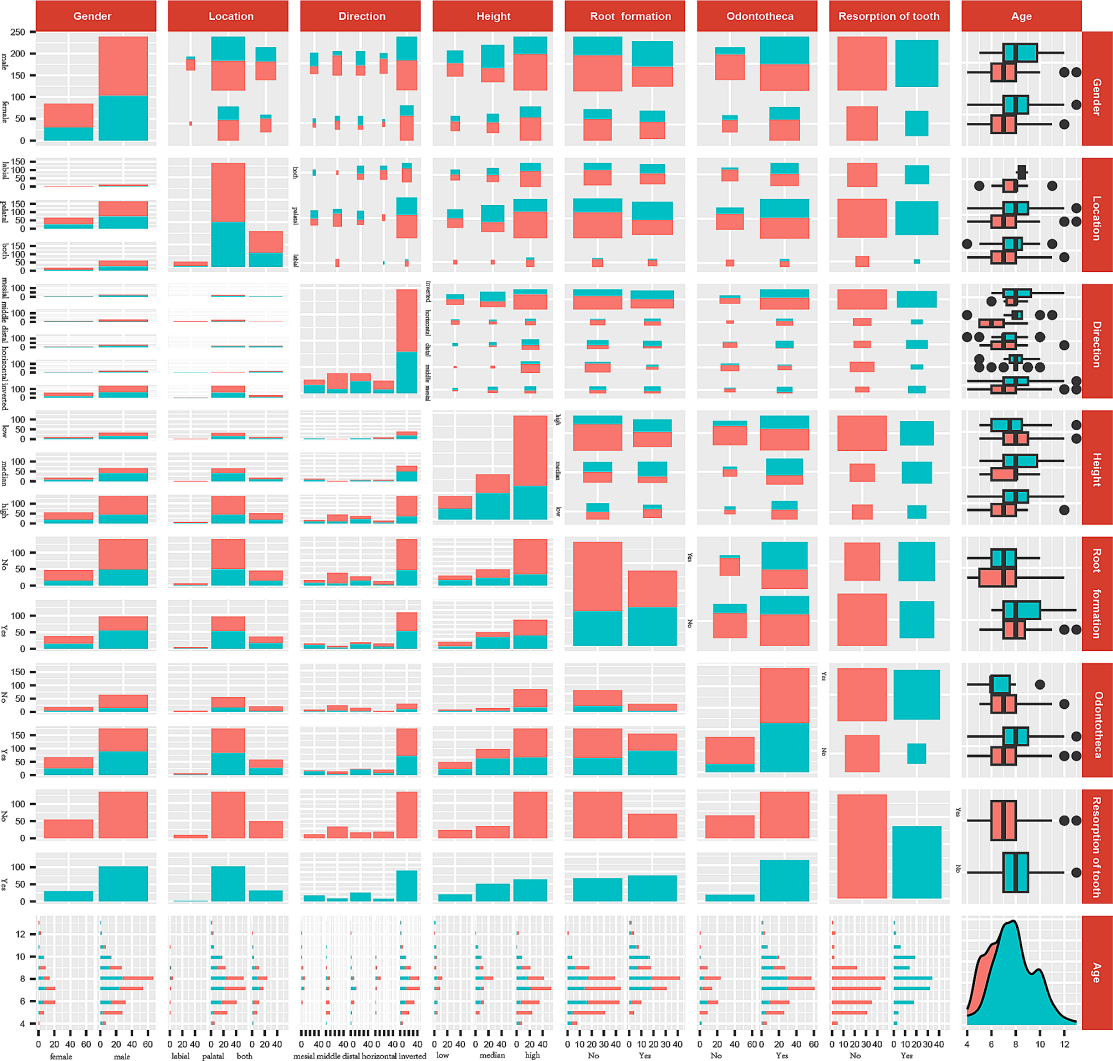
Clinical parameter description of various risk factors and root resorption

The pairwise correlation between each factor and the relationship between each factor and root resorption was shown in (Fig. 2). Age (Z=-4.384, *P* < 0.001), crown direction (χ2 = 18.255, *P* < 0.001), development stage (χ2 = 10.519, *P* < 0.001), impaction height (χ2 = 17.608, *P* < 0.001) and odontotheca (χ2 = 14.502, *P* < 0.001) were significant risk factors (Tables 1 and 2). The null hypothesis of this study was that there was no difference in the distribution of these risk factors between the root resorption group and the root non-resorption group. These results did not support the null hypothesis based on Pearson chi-square test and Wilcox rank sum test.

**Table 2:** Descriptive and inferential statistics of relationship between age and root resorption

	Without root resorption	Root resorption	Z	*p*-value*
Age	191	133	-4.384	0.0001

* Wilcox rank sum test *P*-value (significance at *p* < 0.05)

Predictors then were identified using binary logistic regression (Tables 3 and 4). The goodness-of-fit was determined by using the Hosmer and Lemeshow test, in which a *P* > 0.5 indicated that the model passed the test. Our null hypothesis is that the regression equation coefficients are equal to 0, meaning that these variables have no effect on root resorption. However, at a significance level of 0.05, we can reject the null hypothesis. Two factors remained obviously significant: (1) odontotheca of the supernumerary tooth (*P* < 0.01) with an odds ratio of 2.936 (enlarged/normal); (2) patient’s age (*P* < 0.01) with an odds ratio of 1.261. In addition, combined with clinical experience and the results of binary logistic regression analysis by R language showed that the crown located in the middle and distal directions, supernumerary tooth in the high and median position, and the completion of root development could be included in the regression equation. The logistic regression was logitP = -2.9365 + 0.2322 X ^1^ -0.2418 X ^2^ + 1.4961 X ^3^ + 0.4390 X ^4^ − 0.6414 X ^5^ + 0.0590 X ^6^ + 1.0570 X ^7^ (X^1^ = Patient’s age, X^2^ = Middle direction, X^3^ = Distal direction, X^4^ = Located in the middle third of the root of the adjacent tooth, X^5^ = Located in the neck of adjacent tooth, X^6^ = Stage of root development, X^7^ = Odontotheca). The classification table showed that the model was overall 74.5% correct.

**Table 3:** Correlation coefficients in binary logistic regression equations derived from R language data packets

	Coef	S.E.	Wald Z	*p*-value*
Intercept	-2.9365	0.8462	-3.47	0.0005
Age	0.2322	0.0928	2.50	0.0123
Direction(middle)	-0.2418	0.5600	-0.43	0.6659
Direction(distal)	1.4961	0.4448	3.36	0.0008
Height(median)	0.4390	0.4048	1.08	0.2781
Height(high)	-0.6414	0.3912	-1.64	0.1011
Root formation	0.0590	0.2971	0.20	0.8426
Odontotheca	1.0570	0.3592	2.94	0.0033

Coef = regression coefficient; S.E.=standard error; Wald Z = Wald test; *P-value (significance at *p* < 0.05)

**Table 4:** Binary logistic regression analysis screening variables and their parameter values

		B	S.E.	Wald	df	*p*-value*	Exp(B)	CI	
Age		0.232	0.085	7.449	1	0.006	1.261	1.068	1.490
Direction(mesial = 1)				17.682	4	0.001			
Direction(middle = 2)		0.972	0.619	2.470	1	0.116	0.378	0.112	1.272
Direction(distal = 3)		0.369	0.574	0.413	1	0.520	1.446	0.469	4.459
Direction(horizontal = 4)	-1.638		0.652	6.313	1	0.012	0.194	0.054	0.698
Direction(inverted = 5)		-1.036	0.480	4.660	1	0.031	0.355	0.139	0.909
Height(low = 1)				9.025	2	0.011			
Height(median = 2)		0.479	0.405	1.399	1	0.237	1.614	0.730	3.567
Height(high = 3)		-0.413	0.381	1.180	1	0.277	0.661	0.314	1.394
Root formation (Yes)		0.288	0.280	1.060	1	0.303	1.334	0.771	2.307
Odontotheca (Yes)		1.077	0.341	9.970	1	0.002	2.936	1.505	5.731
Intercept		-2.133	0.893	5.701	1	0.017	0.119		

B = regression coefficient; S.E.= standard error; Wald Z = Wald test; df = degree of freedom; Exp(B) = OR, odds ratio; CI = Confidence Intervals; *P-value (significance at *p* < 0.05)

**Fig. 3:**
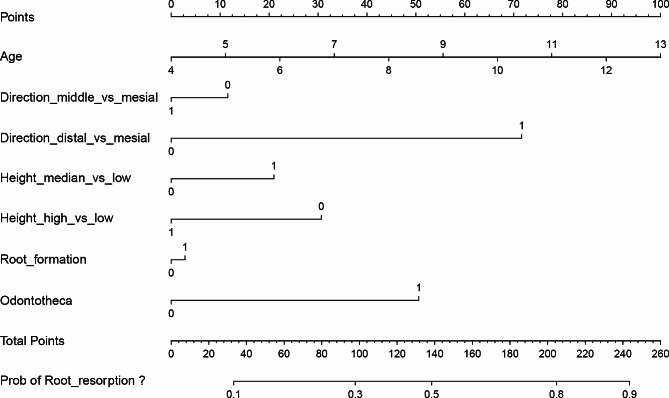
Nomogram for evaluating impacted supernumerary tooth-associated root resorption in children

We used a Nomogram graph to visualize the prediction result(Fig. 3). The Area Under Receiver Operating Characteristic Curve (AUC) was 0.863(95%CI, 0.747–0.979), so the prediction model was reasonably true (see Figure S1). The Decision Curve Analysis (DCA) suggested that this model may better meet the actual needs of clinical decision-making (see Figure S2). The calibration plot revealed good predictive accuracy between the actual probability and predicted probability (see Figure S3).

## Discussion

In this study, we analyse seven variables that are possibly related to ISTARR based on existing literatures.

Crown mesiodistal direction of the impacted tooth may also contribute to the cause of root resorption of adjacent tooth. Inclined orientation of impacted tooth were found to correlate with root resorption of adjacent teeth [18]. Townend et al. [19] reported that a vertically impacted canine increases the risk of adjacent tooth resorption. However, the identification of these significant results reveals only limited information about the covariant relations among the tested variables. In this study, although the individual analysis indicated that the direction of the impacted tooth appeared to be a significant risk factor for root resorption of adjacent tooth, this variable failed to be significant in the logistic regression analysis. As a result, the direction of the impacted tooth may not be a predominant risk factor for ISTARR. Interactions between position of the impacted supernumerary tooth is not respectively significant either.

The depth of the impacted tooth could be related to its eruption trend; the lower impacted tooth has a higher trend, which may increase the risk of root resorption by changing the local environment involving the adjacent root. In our study, the supernumerary tooth impacted in median depth causes a higher ratio of incisor root resorption than supernumerary tooth impacted in low depth, although the trend is not significant (*P* = 0.278).

A previous report [8] showed that the average distance from the impacted tooth cusp tip to the occlusal plane was longer in their adjacent root resorption group. Dental follicles are to protect the tooth during development and eruption. The width of the follicle is the largest distance from the crown of the supernumerary tooth to the periphery of the follicle, and a width greater than 2 mm indicating an enlarged follicle [20]. Such follicles are thought to arise because of autocrine and paracrine factors such as prostaglandins, epidermal growth factor, and transforming growth factor. The width of the follicle is not identified as a significant risk factor in previous work that used two-dimensional periapical and panoramic images and three-dimensional CT images [21–23]. However, the present study analyses three-dimensional CBCT data of impacted tooth and found that the prevalence of root resorption in the enlarged follicle group was 51.56%, compared to the 27.91% in the normal follicle group. This factor emerged as significant in individual analysis and remained so in the logistic regression analysis. Thus, an enlarged follicle is an important risk factor for ISTARR.

Supernumerary tooth developmental stage is another potential risk factor for ISTARR (*P* = 0.001). Logistic regression did not detect any significant interaction between supernumerary tooth developmental stage and other factors. Yan et al. [24] reported a higher prevalence of incisor ISTARR associated with closed-apex impacted tooth could simply reflect a longer history of exposure to the eruptive movement of the impacted tooth. Therefore, the description of closed apexes of impacted supernumerary tooth as a predictive factor for ISTARR may need to be further considered. The effect of patient’s age on root resorption is similar.

There may be other potential factors for root resorption, such as geography, ethnicity, pharmacological interventions, etc. These factors were not included in the study because the patients in this study were all of the same ethnicity and had no other medical conditions [25, 26].

The mechanism of root resorption following impaction and the factors involved in the process are not clear. Most authors have stressed the role of physical pressure of the supernumerary tooth. The mechanisms explaining why physical pressure increases risk of root resorption have not been systematically investigated, either. Because radiographic images cannot reflect cellular and molecular detail, these potential mechanisms remain conjecture until confirmed in future studies.

Root resorption of permanent incisors caused by an impacted supernumerary tooth is an underestimated problem. To avoid worsening of the situation, immediate therapeutic operations usually are needed. The proper treatment of impacted supernumerary tooth depends on patient age and general oral health, type of impaction, presence of spacing and crowding, and associated complications such as resorption of adjacent tooth and cystic degeneration. Treatment includes interceptive treatment, surgical exposure, or extraction of the impacted supernumerary tooth [27–29].

The results of the current study show that using CBCT imaging, we can easily observe the features of impacted supernumerary tooth and adjacent tooth. Since the enlarged follicle is an important risk factor, it is important to pay attention to the size of the follicles during the eruption. Path of eruption of supernumerary tooth is very complicated, preventive measures for position of supernumerary tooth seems not so necessary until at the age of 10 years if the supernumerary tooth still don’t erupt.

## Conclusions

By combining CBCT of supernumerary tooth and patient’s characteristics, statistical methods were used to predict the risk of root resorption of adjacent teeth. The significant predictors we identified and the formula (logitP=-2.9365 + 0.2322 X ^1^ -0.2418 X ^2^ + 1.4961 X ^3^ + 0.4390 X ^4^ − 0.6414 X ^5^ + 0.0590 X ^6^ + 1.0570 X ^7^)(X^1^ = Patient’s age, X^2^ = Middle direction, X^3^ = Distal direction, X^4^ = Located in the middle third of the root of the adjacent tooth, X^5^ = Located in the neck of adjacent tooth, X^6^ = Stage of root development, X^7^ = Odontotheca enlargement) we offered here can help clinicians evaluate the risk of adjacent tooth root resorption. Future studies can collect more data to improve the prediction accuracy and explore possible interceptive treatment approaches for impacted supernumerary tooth-associated root resorption prevention.

### Electronic supplementary material

Below is the link to the electronic supplementary material.

Supplementary Material 1

Supplementary Material 2

Supplementary Material 3

## Data Availability

The datasets generated and/or analyzed during the present study are not publicly available as ethics approval was granted on the basis that only the researchers involved in the study could access the identified data but are available and accessible from the corresponding author on reasonable request.
