# Supplementary material for: Risk prediction model of impacted supernumerary tooth-associated root resorption in children based on cone-beam computed tomography analysis: a case control study
Source: BMC Oral Health. 2024 Aug 9;24:920. doi: 10.1186/s12903-024-04493-2 (PMC11312240; doi:10.1186/s12903-024-04493-2)
Supplement: Supplementary file 1 — Supplementary Material 1 [file 12903_2024_4493_MOESM1_ESM.pdf]

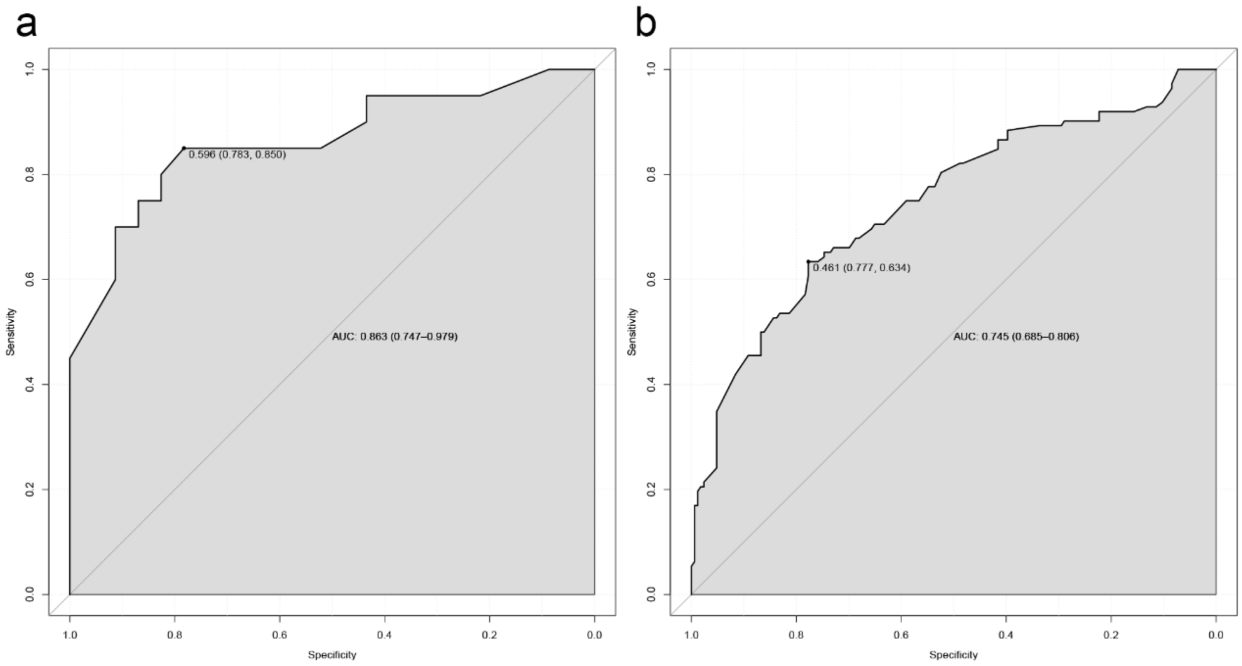
a
b
Sensitivity
Specificity
0.596 (0.783, 0.850)
AUC: 0.863 (0.747–0.979)
0.461 (0.777, 0.634)
AUC: 0.745 (0.685–0.806)

Figure S1

ROC curves estimating the predictive performance of the format constructed in the training and validation cohort.

ROC curve constructed in the training cohort(a). ROC curve constructed in the training cohort(b).
