# Supplementary material for: Risk prediction model of impacted supernumerary tooth-associated root resorption in children based on cone-beam computed tomography analysis: a case control study
Source: BMC Oral Health. 2024 Aug 9;24:920. doi: 10.1186/s12903-024-04493-2 (PMC11312240; doi:10.1186/s12903-024-04493-2)
Supplement: Supplementary file 2 — Supplementary Material 2 [file 12903_2024_4493_MOESM2_ESM.pdf]

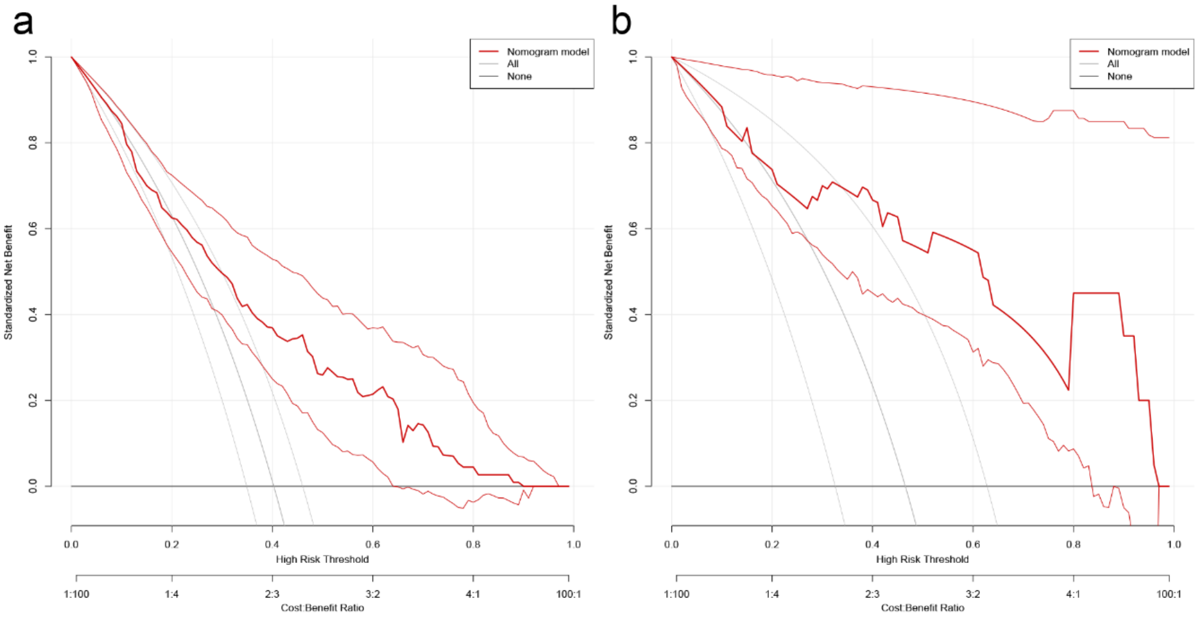

a
b
Nomogram model
All
None
Standardized Net Benefit
High Risk Threshold
Cost:Benefit Ratio
0.0
0.2
0.4
0.6
0.8
1.0
1:100
1:4
2:3
3:2
4:1
100:1

Figure S2

Decision curve analysis (DCA) showing the clinical benefit of column line graphs constructed in the training and validation cohort.

DCA curve constructed in the training cohort(a). DCA curve constructed in the training cohort(b).
