# Supplementary material for: Risk prediction model of impacted supernumerary tooth-associated root resorption in children based on cone-beam computed tomography analysis: a case control study
Source: BMC Oral Health. 2024 Aug 9;24:920. doi: 10.1186/s12903-024-04493-2 (PMC11312240; doi:10.1186/s12903-024-04493-2)
Supplement: Supplementary file 3 — Supplementary Material 3 [file 12903_2024_4493_MOESM3_ESM.pdf]

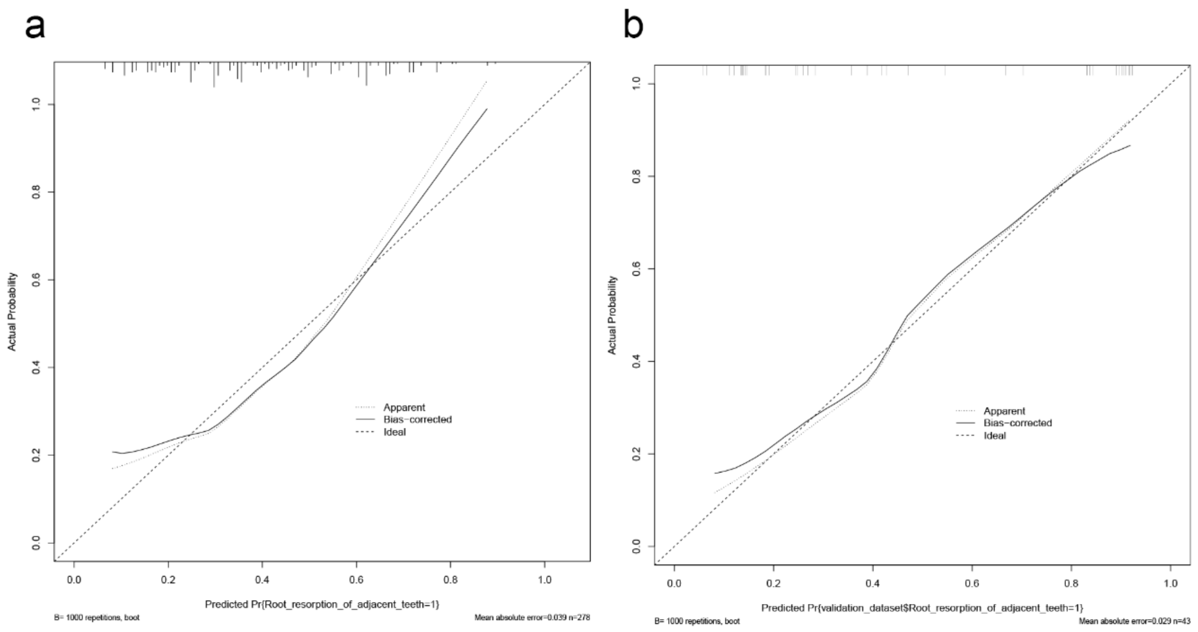
a
b
Actual Probability
Predicted Pr{Root_resorption_of_adjacent_teeth=1}
Apparent
Bias−corrected
Ideal
B= 1000 repetitions, boot
Mean absolute error=0.039 n=278
Predicted Pr{validation_dataset$Root_resorption_of_adjacent_teeth=1}
Mean absolute error=0.029 n=43

Figure S3

Calibration curves to estimate the prediction accuracy of the column line graphs constructed in the training and validation cohort.

Calibration curve constructed in the training cohort(a). Calibration curve constructed in the training cohort(b).
